# AMP-activated protein kinase activation mediates CCL3-induced cell migration and matrix metalloproteinase-2 expression in human chondrosarcoma

**DOI:** 10.1186/1478-811X-11-68

**Published:** 2013-09-18

**Authors:** Chin-Jung Hsu, Min-Huan Wu, Chin-Yuan Chen, Chun-Hao Tsai, Horng-Chaung Hsu, Chih-Hsin Tang

**Affiliations:** 1 School of Chinese Medicine, College of Chinese Medicine, China Medical University, Taichung, Taiwan; 2 Department of Orthopedic Surgery, China Medical University Hospital, Taichung, Taiwan; 3 Graduate Institute of Basic Medical Science, China Medical University, No. 91, Hsueh-Shih Road, Taichung, Taiwan; 4 Department of Medicine and Graduate Institute of Clinical Medical Science, China Medical University, Taichung, Taiwan; 5 Department of Pharmacology, School of Medicine, China Medical University, Taichung, Taiwan; 6 Department of Biotechnology, College of Health Science, Asia University, Taichung, Taiwan; 7 Department of Exercise Health Science, National Taiwan University of Physical Education and Sport, Taichung, Taiwan

**Keywords:** CCL3, Chondrosarcoma, AMPK, MMP-2, CCR5

## Abstract

Chemokine (C-C motif) ligand 3 (CCL3), also known as macrophage inflammatory protein-1α, is a cytokine involved in inflammation and activation of polymorphonuclear leukocytes. CCL3 has been detected in infiltrating cells and tumor cells. Chondrosarcoma is a highly malignant tumor that causes distant metastasis. However, the effect of CCL3 on human chondrosarcoma metastasis is still unknown. Here, we found that CCL3 increased cellular migration and expression of matrix metalloproteinase (MMP)-2 in human chondrosarcoma cells. Pre-treatment of cells with the MMP-2 inhibitor or transfection with MMP-2 specific siRNA abolished CCL3-induced cell migration. CCL3 has been reported to exert its effects through activation of its specific receptor, CC chemokine receptor 5 (CCR5). The CCR5 and AMP-activated protein kinase (AMPK) inhibitor or siRNA also attenuated CCL3-upregulated cell motility and MMP-2 expression. CCL3-induced expression of MMP-2 and migration were also inhibited by specific inhibitors, and inactive mutants of AMPK, p38 mitogen activated protein kinase (p38 or p38-MAPK), and nuclear factor κB (NF-κB) cascades. On the other hand, CCL3 treatment demonstrably activated AMPK, p38, and NF-κB signaling pathways. Furthermore, the expression levels of CCL3, CCR5, and MMP-2 were correlated in human chondrosarcoma specimens. Taken together, our results indicate that CCL3 enhances the migratory ability of human chondrosarcoma cells by increasing MMP-2 expression via the CCR5, AMPK, p38, and NF-κB pathways.

## Introduction

Chondrosarcomas are the third most common bone tumors, after myelomas and osteosarcomas. Chondrosarcomas are rapidly progressive, pathologically diverse, and highly malignant, and to date, surgical resection remains the primary treatment for these sarcomas. They also have the potential for distant metastasis [1]. Therefore, better strategies of treatment will ultimately require understanding of the molecular mechanisms of the metastasis step of human chondrosarcoma and identifying and specifically targeting.

Metastasis is a multistage process that requires cancer cells to escape from the primary tumor, survive in the circulation, seed at distant sites, and grow. Metastasis increases cell motility, induction of vascular and lymphatic angiogenesis, and migration and invasion to other organs [2]. The invasion of tumor cells is a complex, multistage process. To facilitate cell motility, invading cells need to change their cell-cell adhesion properties, rearrange the extracellular matrix (ECM) environment, suppress anoikis, and reorganize their cytoskeleton [3]. MMPs play important roles in these processes because their proteolytic activities assist in the degradation of the ECM and basement membranes [4,5]. In addition to MMPs, cytokines, growth factors, and chemokines have all been shown to regulate tumor cell invasion through autocrine or paracrine pathways [6]. Previous studies have demonstrated the expression of MMP-1, MMP-2, MMP-3, MMP-9, and MMP-13 in human chondrosarcoma cells [7]. Among the all MMP enzymes, MMP-2 (collagenase-2) is particularly interesting because of its role in cartilage degradation via the breakdown of type II collagen, the major collagen component of cartilage. It has also been reported that MMP-2 plays a critical role in ECM turnover and cell-cell interactions as well as metastases of chondrosarcomas [8].

Chemokines are low-molecular weight secretory proteins that can regulate the chemotaxis and the metabolic activity of specific leukocyte subsets. Their production, in general, is stimulated by pro-inflammatory cytokines, growth factors, and by pathogenic stimuli arising in inflammatory tissues. In diseased tissues, different tumor cell types trigger different complex chemokine networks that influence the quality and quantity of immune-cell infiltration and, consequently, the proliferation, survival, spread, and angiogenic response of malignant cells [9]. CCL3, also known as macrophage inflammatory protein 1α (MIP-1α), is a pro-inflammatory cytokine belonging to the CC chemokine subfamily and is a ligand for CCR5, which stimulates chemotactic activities in a variety of immune cells such as monocytes, lymphocytes, macrophages [10,11]. CCL3 has also been implicated in the regulation of cancer cell growth, angiogenesis and metastasis of different tumors such as melanoma [12], colorectal cancer [13], and renal cell carcinoma [14].

Epidemiological studies have shown that energy availability is associated with an increased risk of several metabolic diseases such as obesity, hypertension, diabetes, and induced cancers [15,16]. Aberrant energy metabolism may induce systemic and chronic inflammation both at the cellular and whole-body levels, and hence provide the microenvironment [17]. AMPK is a critical regulator of glucose intake and energy balance and modulates energy regulation involved in glucose and lipid metabolism [18,19]. On the other hand, AMPK has been identified as a novel target in tumor cell migration and invasion [20]. Despite this, the role of AMPK activation in CCL3-mediated cancer migration has not been investigated in chondrosarcomas. In this study, we observed that CCL3 increases the migration of human chondrosarcoma cells and upregulates the expression of MMP-2. Furthermore, we observed that the CCR5 receptor, AMPK, p38, and NF-κB signaling pathways are involved in this process.

## Materials and methods

### Materials

Anti-mouse and anti-rabbit IgG-conjugated horseradish peroxidase, rabbit polyclonal antibodies specific for β-actin, AMPK, phospho(p)-AMPK (Thr^172^), p38, p-p38, inhibitor of kappa B (IκB), p-IκBα, IκB kinase (IKKα/β) (Ser^180/181^), NF-κB p65 subunit (p65), p-p65 (Ser^536^), and MMP-2; control shRNA (sc-108060) and CCL3 shRNA (sc-44722-SH) plasmids were purchased from Santa Cruz Biotechnology (Santa Cruz, CA). AMPK inhibitors (Ara A and compound C), p38 inhibitor (SB203580), IκB protease inhibitor (TPCK), NF-κB inhibitor (PDTC), and MMP-2 inhibitor were purchased from Calbiochem (San Diego, CA). CCL3 and CCR5 monoclonal antibody (mAb) were purchased from Abcam (Cambridge, MA). Met-RANTES was purchased from R&D Systems (Minneapolis, MN, USA). The p38 dominant-negative MAPK mutant was provided by Dr. J. Han (Southwestern Medical Center, Dallas, TX). The IKKα (KM) (K44A) and IKKβ (KM) (K44A) mutants were gifted by Dr. H. Nakano (Juntendo University, Tokyo, Japan). All other chemicals were purchased from Sigma-Aldrich (St. Louis, MO).

### Cell cultures

The human chondrosarcoma cell line (JJ012) was kindly provided by the laboratory of Dr. Sean P. Scully (University of Miami School of Medicine, Miami, FL) and originated from Dr. Joel Block (Rush University Medical Center, Chicago, Illinois). JJ012 cells were cultured in a complete medium containing Dulbecco’s modified Eagle’s medium (DMEM)/α-minimum essential medium (α-MEM) supplemented with 10% fetal bovine serum (FBS). The human chondrosarcoma cell line (SW1353) was obtained from the American Type Culture Collection. SW1353 cells were cultured in a complete medium containing DMEM supplemented with 10% FBS. All experiments with cells were maintained at 37°C in a humidified atmosphere of 5% CO_2_.

CCL3 shRNA or control shRNA plasmids were transfected into JJ012 cells by using the transfection reagent, Lipofectamine 2000. At 24 h after transfection, stable transfectants were selected with 10 μg/mL puromycin (Life Technologies). The selection medium was replaced every 3 days and 2 weeks after selection, and puromycin-resistant cells and their clones were isolated.

### Migration and invasion assay

The migration assay was performed using the Transwell assay (Costar, Acton, MA; pore size, 8-μm) in 24-well dishes. For the invasion assay, filters were precoated with 30 μL Matrigel Basement Membrane Matrix (BD Biosciences, Bedford, MA) for 30 min. The procedure for both migration and invasion assays was as follows. Before the migration assay was performed, cells were pretreated for 30 min with different concentrations of inhibitors, including the CCR5 mAb, compound C, Ara A, SB208530, TPCK, PDTC, MMP-2 inhibitor, or vehicle control (0.1% DMSO). Approximately 1 × 10^4^ cells in 100 μL of serum-free medium was placed in the upper chamber, and 300 μL of the same medium containing CCL3 was placed in the lower chamber. The plates were incubated for 24 h at 37°C in 5% CO_2_, and cells were then fixed in methanol for 15 min and stained with 0.05% crystal violet in PBS for 15 min. Cells on the upper side of the filters were removed with cotton-tipped swabs, and the filters were washed with PBS. Cells on the underside of the filters were examined and counted under a microscope. Each clone was plated in triplicate in each experiment, and each experiment was repeated at least 3 times [3,21].

### Wound-healing migration assay

For wound-healing migration assays, cells were seeded on 12-well plates at a density of 1 × 10^5^ cells/well in culture medium. At 24 h after seeding, the confluent monolayer of culture was scratched with a fine pipette tip, and migration was visualized by microscopy. The rate of wound closure was observed at the indicated times [22].

### Zymographic analysis

Supernatants collected from JJ012 cell cultures were mixed with sample buffer without reducing agents or heating. Samples were loaded onto a 10% SDS-PAGE gel containing 1 mg/ml gelatin and electrophoresed under constant voltage. Subsequently, the gel was washed with 2.5% Triton X-100 to remove SDS, rinsed with 50 mM Tris–HCl, pH 7.5, and then incubated overnight at room temperature with a developing buffer (50 mM Tris–HCl, pH 7.5, 5 mM CaCl_2_, 1 μM ZnCl_2_, 0.02% thimerosal, and 1% Triton X-100). Zymographic activity was revealed by staining with 1% Coomassie Blue.

### Transfection of siRNAs or mutants

ON-TARGETplus siRNA targeting AMPKα1, AMPKα2 (The two catalytic subunit of AMPK; transfection of cells with AMPKα1 or AMPKα2 siRNA inhibited AMPKα1 or AMPKα2 expression, respectively; Additional file 1: Figure S1), MMP-2, and controls were purchased from Dharmacon Research (Lafayette, CO, USA). Transient transfection of siRNAs (10 nM) or dominant-negative mutants (0.5 μg) was carried out using DharmaFECT1 transfection reagent or Lipofectamine 2000 (Invitrogen, Carlsbad, CA), according to the manufacturer’s instructions, respectively.

### Quantitative real-time polymerase chain reaction

Quantitative real-time polymerase chain reaction (qPCR) analysis was carried out using TaqMan® one-step PCR Master Mix (Applied Biosystems, Foster City, CA, USA). Total complementary DNA (100 ng/25 μL reaction) was mixed with sequence-specific primers and TaqMan® probes according to the manufacturer’s instructions. All target gene primers and probes were purchased commercially, and GAPDH was used as the internal control (Applied Biosystems). qPCR assays were carried out in triplicate with a StepOnePlus sequence detection system. The cycling conditions were 10 min polymerase activation at 95°C followed by 40 cycles at 95°C for 15 s and 60°C for 60 s. To calculate the cycle number at which the desired transcript was detected (denoted *C*_T_), the threshold was set above the non-template control background and within the linear phase of target gene amplification.

### Western blot analysis

Cell lysates were prepared as described previously [23]. Proteins were resolved by SDS-PAGE and transferred to polyvinyldifluoride (PVDF) membranes (Immobilon). The blots were blocked with 4% BSA for 1 h at room temperature and then probed with rabbit anti-human antibodies against MMP-2, p-IKKα/β, IKKα/β, p65, p-p65, p-AMPK, AMPK, p-p38, or p38 (1:1000) each separately for 1 h at room temperature. After three washes, the blots were incubated with peroxidase-conjugated donkey anti-rabbit secondary antibody (1:1000) for 1 h at room temperature. The blots were visualized by enhanced chemiluminescence using Kodak X-OMAT LS film (Eastman Kodak, Rochester, NY, USA).

### Chromatin immunoprecipitation assay

Chromatin immunoprecipitation analysis was performed as described previously [24]. DNA immunoprecipitated using the anti-p65 antibody was purified. The DNA was then extracted with phenol-chloroform. The purified DNA pellet was subjected to PCR. PCR products were resolved by 1.5% agarose gel electrophoresis and visualized by UV. The primers 5′-CCCCTGTTCAAGATGGAGTC-3′ and 5′-CCCAGGTTGCTTCCTTACCT-3′ were utilized in the PCR to amplify the human MMP-2 promoter region (−673 to −517) [25].

### Reporter assay

Human chondrosarcoma cells were transfected with a reporter plasmid using Lipofectamine 2000 (Invitrogen) according to the manufacturer’s recommendations. At 24 h after transfection, the cells were pretreated with inhibitors for 30 min, and then, CCL3 or vehicle was added for 24 h. Cell extracts were then prepared, and luciferase and β-galactosidase activities were measured [3].

### Immunofluorescence staining

Human chondrosarcoma cells were plated on 24-well culture plates with coverslips. Cells were treated with CCL3 (30 ng/ml) and washed twice with ice-cold phosphate-buffered saline. Immunofluorescence staining using a primary anti-p65 monoclonal antibody was performed as described previously [22].

### Immunohistochemistry

The human chondrosarcoma tissue array was purchased from Biomax (Rockville, MD, USA; 8 cases for normal cartilage, 4 cases for type Ib chondrosarcoma, and 4 cases for type IIb chondrosarcoma). The tissues were placed on glass slides, rehydrated, and incubated in 3% hydrogen peroxide to block the endogenous peroxidase activity. After trypsinization, sections were blocked by incubation in 3% bovine serum albumin (BSA) in PBS. The slides were incubated at 4°C overnight with a 1:50 diluted primary antibody, which was either monoclonal mouse anti-human CCL3, CCR5, or MMP-2 antibody. After being washed 3 times with PBS, samples were treated with goat anti-mouse IgG biotin-labeled secondary antibodies at a dilution of 1:50. The bound antibodies were detected with an ABC kit (Vector Laboratories, Burlingame, CA). The slides were stained with the chromogen diaminobenzidine, washed, counterstained with Delafield’s hematoxylin, dehydrated, treated with xylene, and mounted. The intensity of staining was evaluated as 0, 1+, 2+, 3+, 4+, and 5+ for no staining, very weak staining, weak staining, moderate staining, strong staining, and very strong respectively. IHC score was determined as the sum of the intensity score.

### Statistics

Data are presented as mean ± standard error of the mean (SEM). Statistical comparison of two groups was performed using the Student’s *t* test. Statistical comparisons of more than two groups were performed using one-way analysis of variance with Bonferroni’s post-hoc test. Analyzing patterns of staining in immunohistochemical studies statistical comparison of two tissue scores was performed using the Regression Analysis Method. In all cases, *p* < 0.05 was considered significant.

## Results

### Correlation of CCL3, CCR5, and MMP-2 expression in human chondrosarcoma specimens

**Figure 1:**
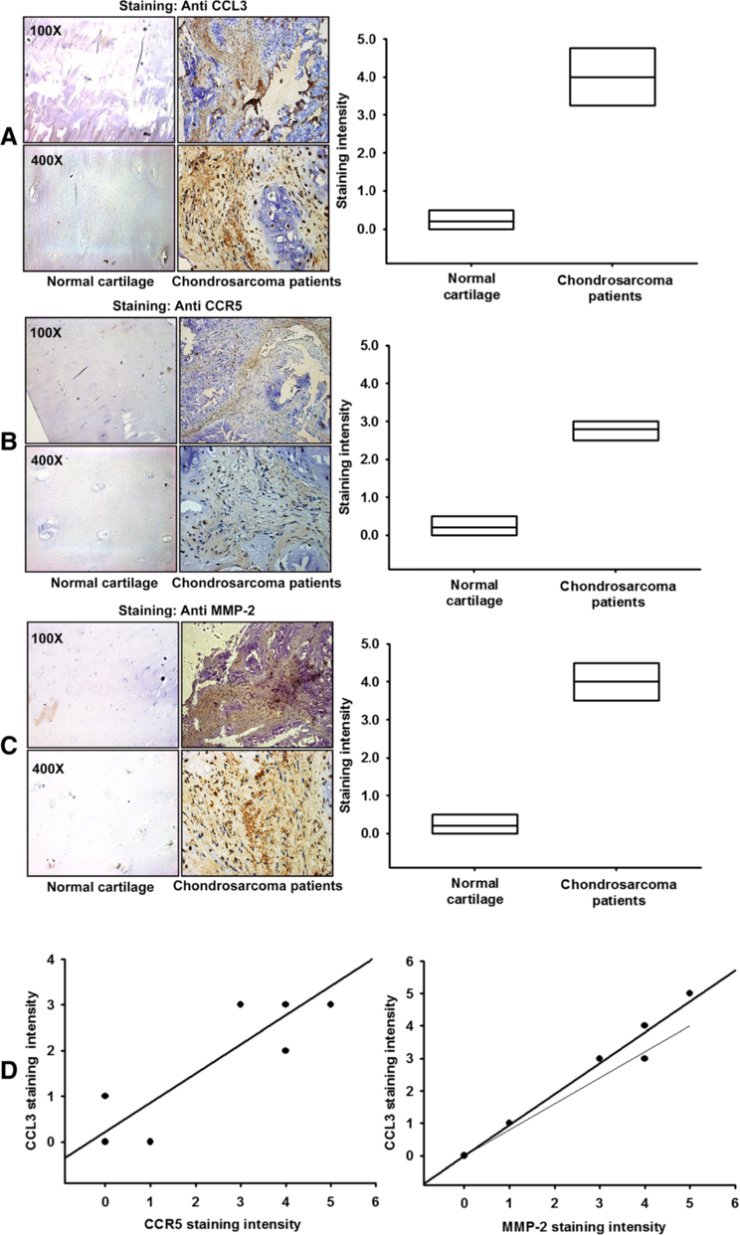
**CCL3, CCR5, and MMP-2 expression correlates with the human chondrosarcoma specimens.** Immunohistochemistry of CCL3 **(A)**, CCR5 **(B)**, and MMP-2 **(C)** expression in normal cartilage and chondrosarcoma tissues. The correlative and quantitative data are shown in **(D)**.

Previous study suggest that CCL3 is overexpressed in many cancer types [26]. However, the expression of CCL3, CCR5 and MMP-2 in chondrosarcoma is still unknown. Therefore, we analyzed samples from chondrosarcoma specimens by immunohistochemical staining. The expression of CCL3, CCR5, and MMP-2 in chondrosarcoma specimens was significantly higher than that in normal cartilage (Figure 1A-C). We also used western blotting to confirm the results from immunohistochemistry that the expression of CCL3 and MMP-2 in chondrosarcoma was also significantly higher than that in chondrocytes (Additional file 1: Figure S2). In addition, the high level of CCL3 expression correlated strongly with CCR5 and MMP-2 expression, which was also indicated by the quantitative data (Figure 1D). Taken together, these results indicate that CCL3, CCR5, and MMP-2 expression are correlated in human chondrosarcoma specimens.

### Involvement of MMP-2 in CCL3-induced migration of chondrosarcoma cells

**Figure 2:**
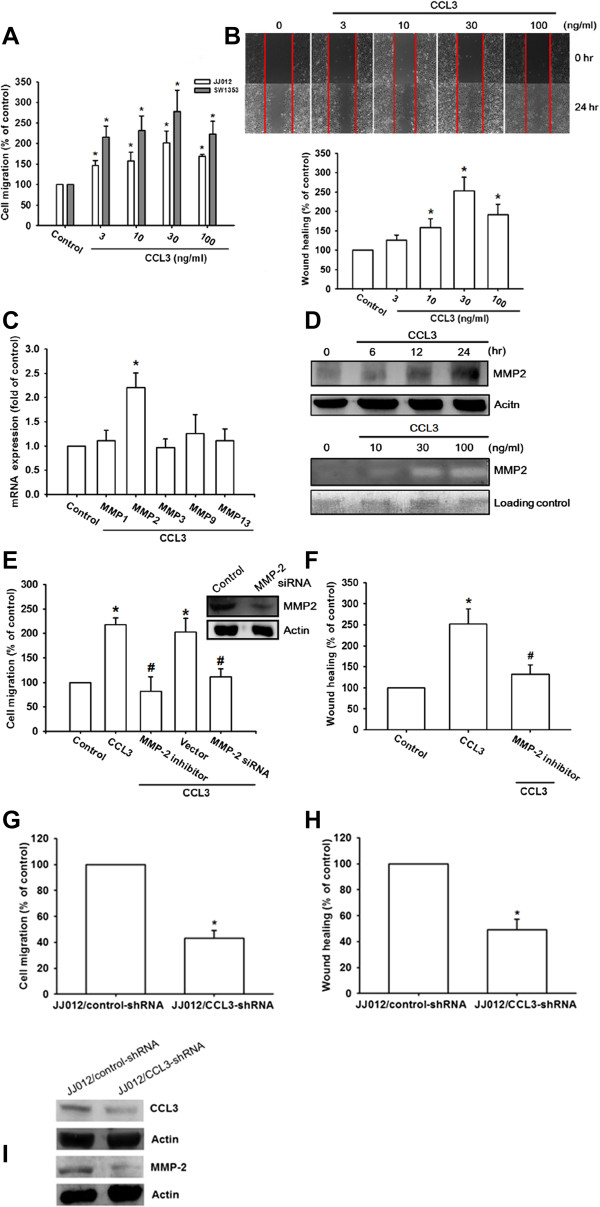
**CCL3-directed migration activity of human chondrosarcoma cells involves up-regulation of MMP-2. (A-B):** Cells were incubated with CCL3 for 24 h, and *in vitro* migration was measured either by Transwell **(A)** or by a wound-healing assay **(B)**. **(C):** JJ012 cells were incubated with CCL3 for 24 h, and MMP-1, MMP-2, MMP-3, MMP-9, and MMP-13 mRNA levels were determined using qPCR. **(D):** JJ012 cells were incubated with CCL3 for the indicated periods or with the indicated doses of CCL3, and cell lysates and supernatants were collected. MMP-2 protein levels in cell lysates and enzymatic activity in supernatants were determined by western blotting and zymography. **(E-F):** JJ012 cells were transfected with MMP-2 siRNA or were pre-treated with MMP-2 inhibitor, and in vitro migration was measured with the Transwell **(E)** or the wound-healing assay **(F)**. **(G-H): ***In vitro* migration activity of JJ012/control-shRNA and JJ012/CCL3-shRNA cells was measured with Transwell and wound-healing assays. **(I):** The protein levels of CCL3 and MMP-2 of in JJ012 cells transfected with control-shRNA or CCL3-shRNA was measured by western blotting. The results are expressed as the mean ± SE. *P < 0.05 compared with control. ^#^*P* < 0.05 compared with CCL3-treated group.

It has been reported that CCL3 promotes tumor metastasis [26]. We therefore directly examined migration in human chondrosarcoma cell lines in response to CCL3 by using Transwell and wound-healing migration assays. CCL3 (3–100 ng/ml) induced cell migration and wound healing migration of chondrosarcoma cells (JJ012 & SW1353) in a concentration-dependent manner (Figure 2A&B). On the other hand, incubation of primary chondrocytes with CCL3 did not induce cell migration (Additional file 1: Figure S3). We further examine whether the JJ012 cells expressed high level of CCL3. Indeed, we found that JJ012 expressed high level of CCL3 than primary chondrocytes (Additional file 1: Figure S4).

Previous studies have shown significant expression levels of MMP-1, -2, -3, -9, and -13 in human chondrosarcoma cells [7,27]. We therefore hypothesized that MMPs may be involved in CCL3-induced chondrosarcoma migration. Incubation of cells with CCL3 increased transcriptional expression of MMP-2 but not other MMPs, as measured by qPCR (Figure 2C). In JJ012 cells, CCL3 increased the expression of MMP-2 protein in a time-dependent manner (Figure 2D; upper panel). MMP-2 protein expression was also increased in the supernatant, and its enzyme activity was upregulated (Figure 2D; lower panel). To examine whether MMP-2 was involved in CCL3-induced cell migration, both the MMP-2 inhibitor and siRNA against MMP-2 were used. Pretreatment of cells with the MMP-2 inhibitor or transfection with MMP-2 specific siRNA abolished CCL3-induced cell migration and would healing activity (Figure 2E&F). To confirm that CCL3 mediates cell migration and MMP-2 expression in human chondrosarcoma cells, JJ012 cells lines stably expressing CCL3 shRNA were established. CCL3 expression in stable transfectants was compared with that in controls by western blotting. The expression of CCL3 was dramatically inhibited in JJ012/CCL3 shRNA cells (Figure 2I). While the knockdown of CCL3 did not affect JJ012 cell growth (data not shown), the migratory ability of JJ012 cells was inhibited (Figure 2G&H) as evaluated using Transwell assays and wound healing migration assays. In addition, CCL3 knockdown also reduced MMP-2 expression in JJ012 cells (Figure 2I). These results indicate that CCL3 upregulates MMP-2 and migration in chondrosarcoma cells, but not necessarily MMP-2 associated.

### CCL3-mediated MMP-2 expression and cell migration are via the CCR5 receptor

**Figure 3:**
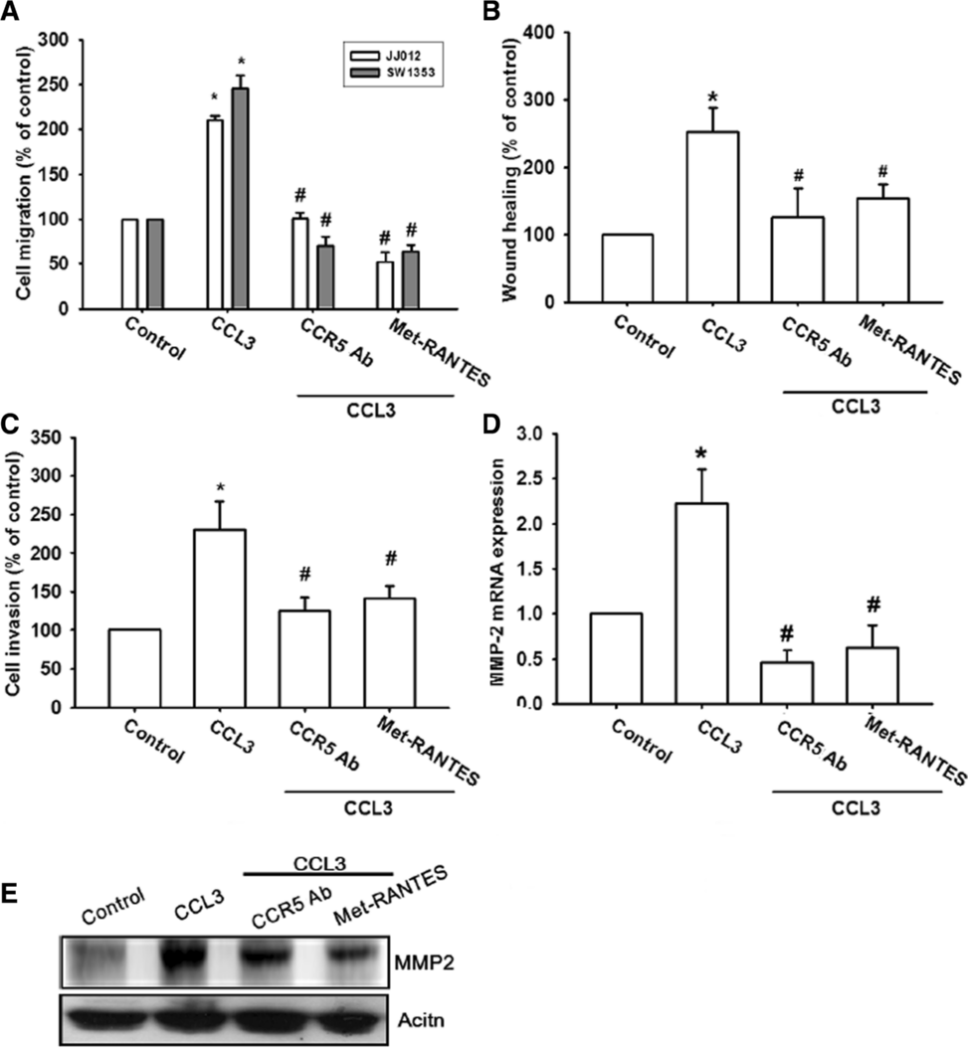
**CCL3 increases cell migration through the CCR5 receptor. (A-E):** Cells were pretreated for 30 min with CCR5 mAb or Met-RANTES. Subsequently, they were stimulated with CCL3 (30 ng/ml) for 24 h, and *in vitro* migration **(A&B)**, invasion **(C)**, and MMP-2 **(D&E)** expression were measured using the Transwell assay, wound healing assay, qPCR, and western blotting. The results are expressed as the mean ± SE. *P < 0.05 compared with control. ^#^*P* < 0.05 compared with CCL3-treated group.

A previous study showed that CCL3 affects cell migration through binding to the cell surface CCR5 receptor [28]. Pretreatment of cells with CCR5 mAb or the CCR5 receptor inhibitor (Met-RANTES) reduced CCL3-induced cell migration and invasion (Figure 3A-C) and inhibited CCL3-induced the mRNA and protein expression of MMP-2 (Figure 3D&E). Therefore, blocking CCR5 reduced CCL3-mediated cell migration and MMP-2 expression in human chondrosarcoma cells.

### The AMPK-dependent p38 pathway is involved in the CCL3-mediated cell migration and MMP-2 expression in chondrosarcoma cells

**Figure 4:**
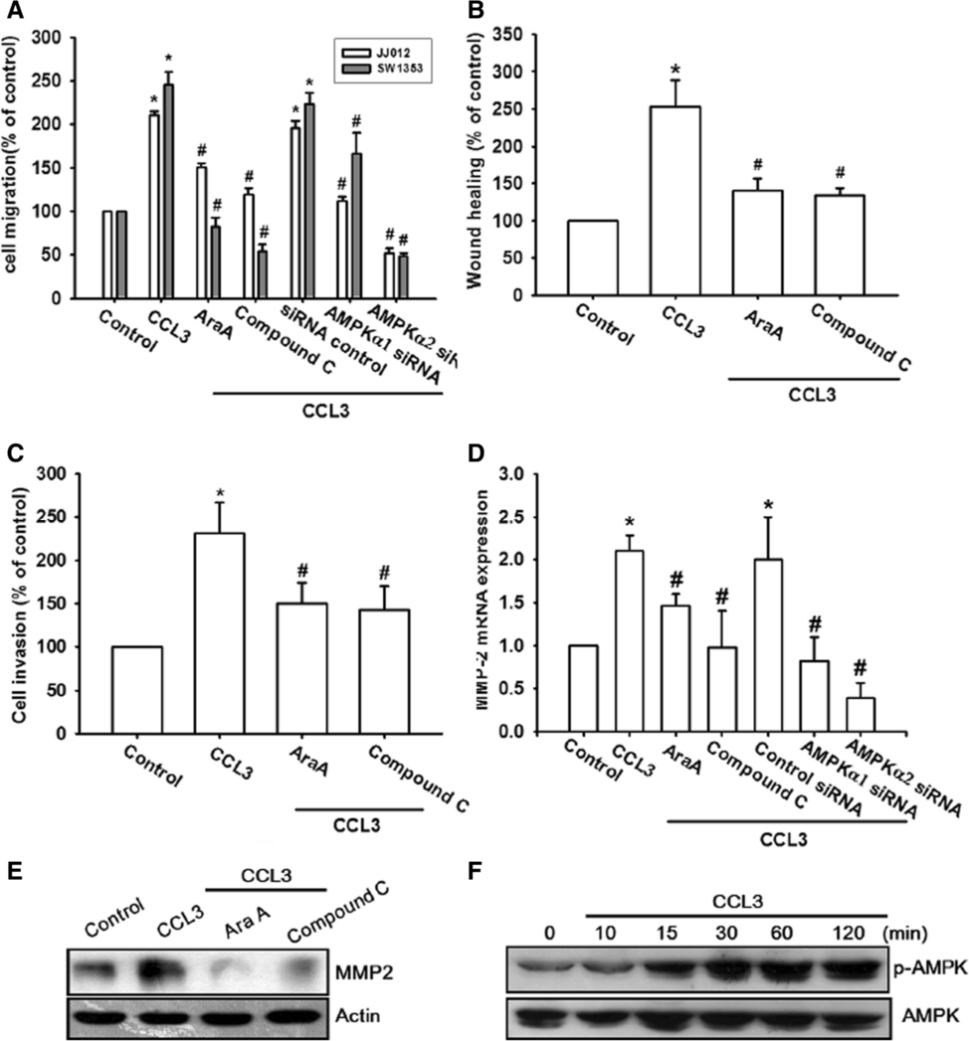
**AMPK pathway mediates CCL3-induced migration in human chondrosarcoma cells. (A-E):** Cells were pretreated for 30 min with Ara A (0.5 mM) and compound C (10 μM) or transfected with AMPKα1 and AMPKα2 siRNA for 24 h, following which they were stimulated with CCL3 (30 ng/ml) for 24 h, and *in vitro* migration **(A&B)**, invasion **(C)**, and MMP-2 **(D&E)** expression were measured with the Transwell assay, wound healing assay, qPCR, and western blotting. **(F):** JJ012 cells were incubated with CCL3 for indicated time intervals, and p-AMPK expression was examined by western blotting. Results are expressed as the mean ± SE. *P < 0.05 compared with control. ^#^*P* < 0.05 compared with CCL3-treated group.

AMPK activation has been reported to mediate tumor cell migration [4,29]. We therefore used AMPK inhibitors (Ara A and compound C) to examine whether AMPK mediates CCL3-induced cell motility. Pretreatment of cells for 30 min with Ara A (0.5 mM) and compound C (10 μM) markedly attenuated CCL3-induced migration, invasion, and MMP-2 expression (Figure 4A-E). To determine the identity of the catalytic subunit of AMPK that mediates CCL3 signaling in human chondrosarcomas, we performed a migration assay by using cells transfected with siRNA specific for either AMPKα1 or AMPKα2. Transfection of cells with either AMPKα1 or AMPKα2 siRNA antagonized CCL3-induced cell migration and MMP-2 expression (Figure 4A&D). The data therefore suggest that AMPKα1 and AMPKα2 are involved in CCL3-mediated migration activity and MMP-2 expression in chondrosarcoma. Furthermore, incubation of cells with CCL3 promoted AMPKα phosphorylation at Thr^172^ in a time-dependent manner (Figure 4F).

**Figure 5:**
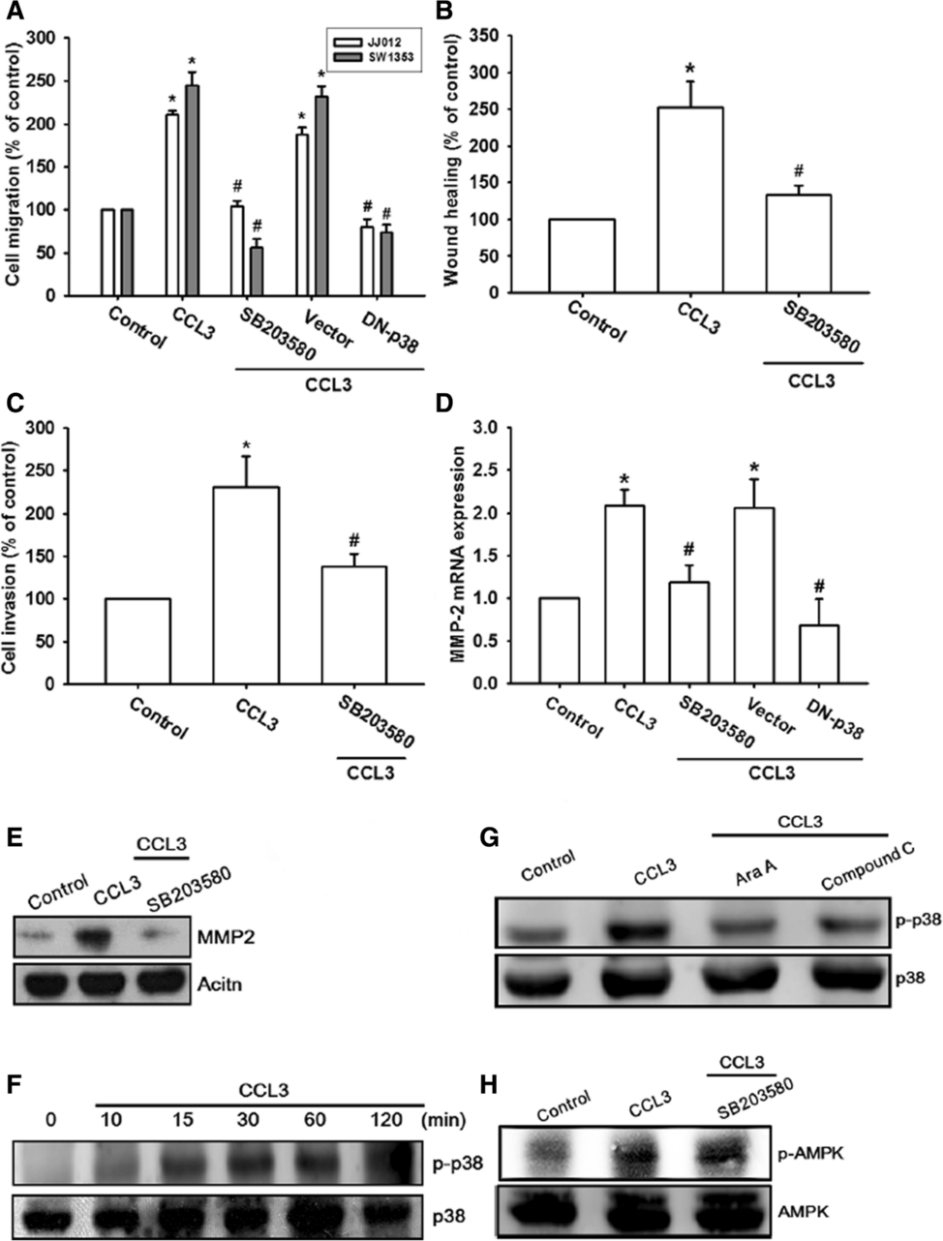
**AMPK-dependent p38 pathway is involved CCL3-induced migration and MMP-2 expression. (A-E):** Cells were pretreated for 30 min with SB203580 (10 μM) or transfected with p38 mutant for 24 h. Subsequently, they were stimulated with CCL3 (30 ng/ml) for 24 h, and *in vitro* migration **(A&B)**, invasion **(C)**, and MMP-2 **(D&E)** expression were measured with the Transwell assay, wound healing assay, qPCR, and western blotting. **(F):** JJ012 cells were incubated with CCL3 for indicated time intervals, and p-AMPK expression was examined by western blotting. **(G&H):** Cells were pretreated for 30 min with Ara A, compound C, or SB203580 followed by stimulation with CCL3. The p-p38 **(G)** and p-AMPK **(H)** expression was measured by western blotting. Results are expressed as the mean ± SE. *P < 0.05 compared with control. ^#^*P* < 0.05 compared with CCL3-treated group.

AMPK-dependent p38 activation has been reported to be involved in the metastasis of human chondrosarcoma [30]. We investigated the role of p38 in mediating CCL3-induced migration by using the specific p38 inhibitor SB203580. Pretreatment of cells with SB203580 (10 μM) or transfection of cells with the p38 mutant abolished CCL3-induced migration, invasion, and MMP-2 expression (Figure 5A-E). In addition, treatment of the chondrosarcoma with CCL3 resulted in time-dependent phosphorylation of p38 (Figure 5F). Moreover, pretreatment of cells with Ara A or compound C for 30 min markedly inhibited CCL3-induced p38 phosphorylation (Figure 5G). In contrast, pretreatment with SB203580 did not effect CCL3-mediated AMPK phosphorylation (Figure 5H). Therefore, these results indicate that p38 may function as a signaling molecule downstream of AMPK in CCL3-mediated cell migration and MMP-2 expression.

### NF-κB signaling pathway is involved in CCL3-mediated MMP-2 up-regulation and migration activity

**Figure 6:**
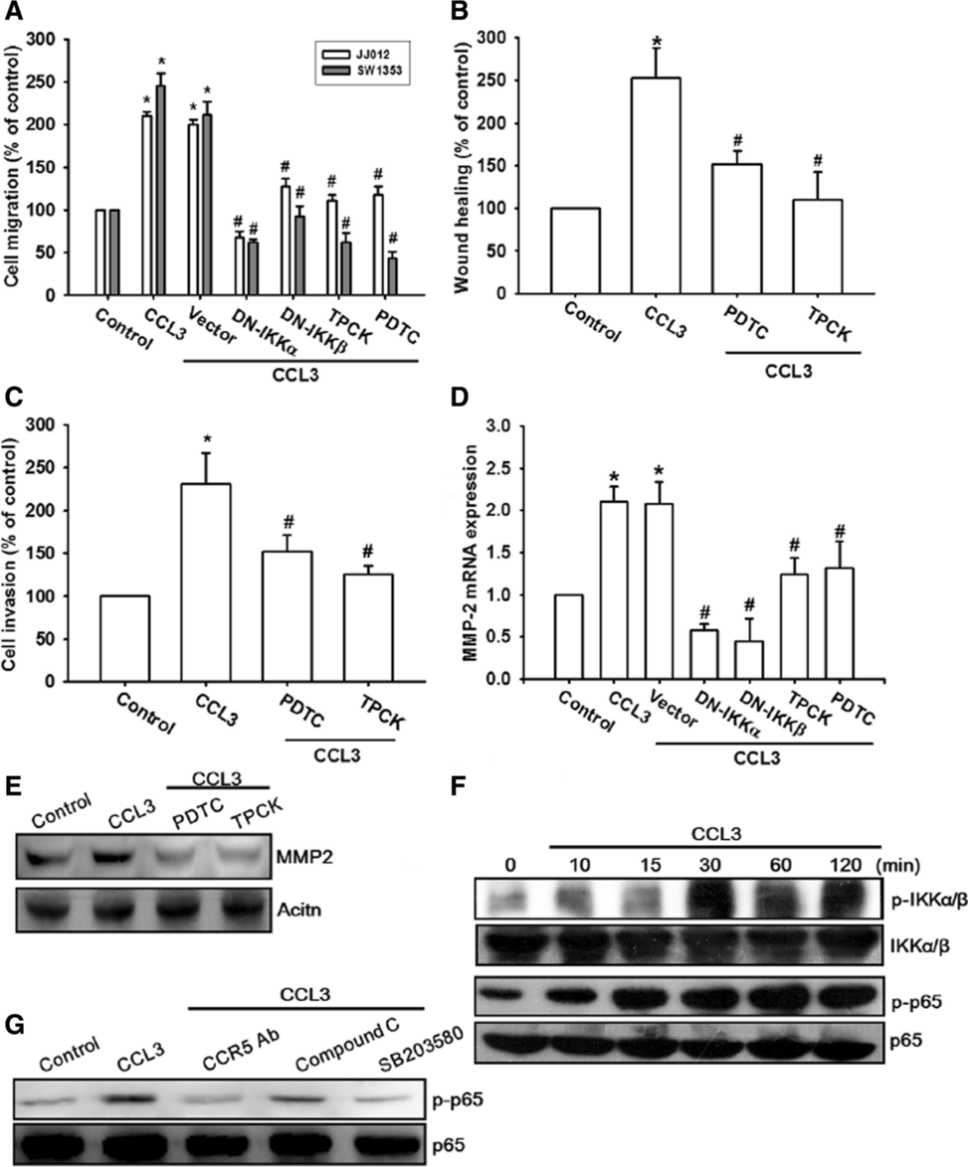
**CCL3 induces cells migration and MMP-2 upregulation through NF-κB. (A-E):** Cells were pretreated for 30 min with PDTC (10 μM) and TPCK (3 μM) or transfected with IKKα and IKKβ mutant for 24 h following which they were stimulated with CCL3 (30 ng/ml) for 24 h, and *in vitro* migration **(A&B)**, invasion **(C)**, and MMP-2 **(D&E)** expression were measured with the Transwell assay, wound healing assay, qPCR, and western blotting. **(F):** JJ012 cells were incubated with CCL3 for indicated time intervals, and p-IKK or p-p65 expression was examined by western blotting. **(G):** Cells were pretreated for 30 min with CCR5 mAb, compound C, or SB203580, and subsequently stimulated with CCL3. The p-p65 expression was measured by western blotting. Results are expressed as the mean ± SE. *P < 0.05 compared with control. ^#^*P* < 0.05 compared with CCL3-treated group.

CCL3 has been reported to induce cell migration through NF-κB activation [31]. To examine whether NF-κB activation was involved in CCL3-induced cell migration, an NF-κB inhibitor, PDTC, or the IκB protease inhibitor, TPCK, were used. Figure 6A-E shows that pre-treatment of cells with either PDTC or TPCK inhibited CCL3-induced migration, invasion, and MMP-2 expression in chondrosarcoma cells. Therefore, CCL3 increased cell migration and MMP-2 expression through NF-κB pathway. We also examined the upstream molecules involved in CCL3-induced NF-κB activation. Stimulation of cells with CCL3 induced IKKα/β phosphorylation in a time-dependent manner (Figure 6F). In addition, transfection with the IKKα or IKKβ mutants markedly reduced CCL3-induced cell motility and MMP-2 expression (Figure 6A&D). These data suggest that activation of IKKα/β was involved in CCL3-induced cell motility of human chondrosarcoma cells. Treatment of JJ012 cells with CCL3 for various periods also resulted in p65 phosphorylation (Figure 6F). Pre-treatment of cells with CCR5 mAb, compound C, or SB203580 reduced CCL3-induced p65 phosphorylation (Figure 6G).

**Figure 7:**
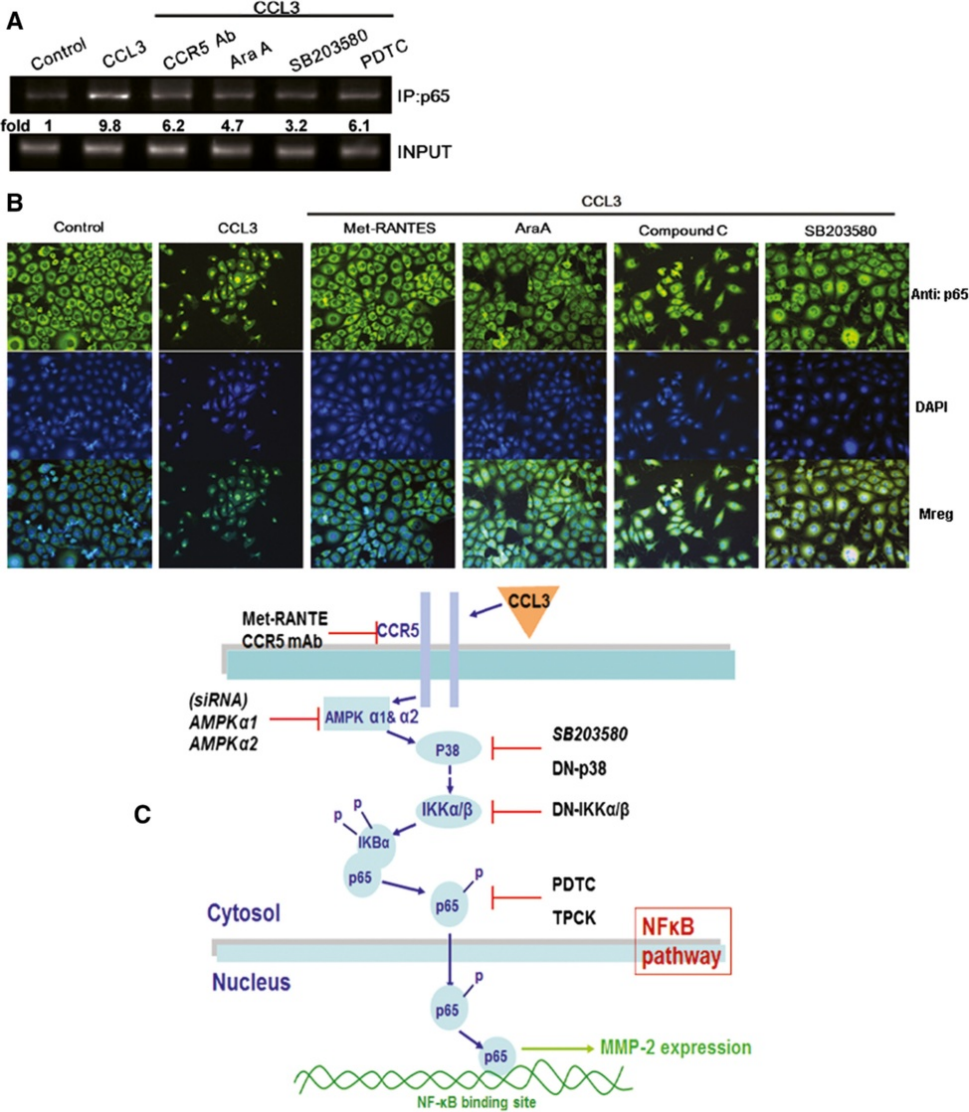
**The CCR5/AMPK/p38 pathway is involved in CCL3-mediated NF-κB activation. (A):** JJ012 cells were pre-treated with CCR5 mAb, Ara A, SB203580, or PDTC followed by stimulation with CCL3 for 120 min and analyses using the chromatin immunoprecipitation assay. Chromatin was immunoprecipitated with anti-p65 antibody. One percentage of the precipitated chromatin was assayed to verify equal loading (input). **(B):** JJ012 cells were pretreated with Met-RANTES, Ara A, compound C, or SB203580 followed by stimulation with CCL3 for 120 min, and p65 immunofluorescence staining was performed. *P < 0.05 compared with control. ^#^*P* < 0.05 compared with CCL3-treated group. **(C):** Schematic diagram of the signaling pathways involved in CCL3-mediated enhanced cell migration in human chondrosarcoma cells. CCL3 increases MMP-2 expression and cell migration through the CCR5 receptor, AMPK, p38, and NF-κB signaling pathways.

Next we examine whether CCR5, AMPK, and p38 are upstream molecules in CCL3-mediated NF-κB activation. CCL3 stimulation increased p65 binding to the NF-κB element on the MMP-2 promoter by using chromatin immunoprecipitation assay (Figure 7A). This was attenuated by pretreatment of cells with CCR5 mAb, Ara A, SB203580, or PDTC (Figure 7A). In addition, Met-RANTES, Ara A, compound C, or SB203580 also reduced CCL3-induced p65 translocation into the nucleus and NF-κB-luciferase activity (Figure 7B & Additional file 1: Figure S5A). On the other hand, co-transfection with p38, IKKα, or IKKβ mutant and AMPKα1 or AMPKα2 siRNA abolished CCL3-induced NF-κB luciferase activity (Additional file 1: Figure S5B). Taken together, these data suggest that activation of the CCR5 receptor, AMPK, and p38 are required for CCL3-induced NF-κB activation in human chondrosarcoma cells.

## Discussion

With the advent of systemic chemotherapy in the management of mesenchymal malignancies such as osteosarcoma and Ewing’s sarcoma, there has been a dramatic increase in the long-term survival of patients. In contrast, chondrosarcomas continue to have a poor prognosis owing to the absence of an effective adjuvant therapy [32]. Chondrosarcoma shows a predilection for metastasis to the lungs and hence it is important to investigate the potential targets for preventing chondrosarcoma metastasis.

Immunohistochemical analyses revealed that the expression of CCL3 in chondrosarcoma patients was higher than that in normal cartilages. In this study, we hypothesized and investigated the paradigm that CCL3 may direct the metastasis of chondrosarcomas. Direct administration of exogenous CCL3 promoted cell migration, invasion, and wound healing activity in chondrosarcoma cells. On the other hand, CCL3-induced MMP-2 expression and cell motility were abolished by CCL3 shRNA. Our data suggest that CCL3 increases MMP-2 expression and subsequently promotes cell migration in human chondrosarcoma cells. The mechanisms underlying CCL3-induced increase in MMP-2 production and cell migration are activation of the CCR5 receptor, AMPK, p38, and NF-κB pathways.

As shown in previous studies, the CCR5 receptor is present on the surface of tumor cells and is responsible for CCL3-mediated cell motility [33]. In this study, immunohistochemical staining revealed that high levels of CCL3 expression strongly correlated with CCR5 expression in human chondrosarcoma patients. Pre-treatment of chondrosarcoma cells with CCR5 mAb or inhibitor blocked CCL3-induced cell migration and reduced MMP-2 expression. Therefore, the CCL3-CCR5 interaction mediated the migratory activity in human chondrosarcoma cells.

Metastasis occurs in multiple steps. Tumor cells degrade the basement membrane, mainly through the use of MMPs [34]. In human cancer cells, MMP-1, MMP-2, MMP-3, MMP-9, and MMP-13 have been found to be correlated with malignant grade and metastasis [4,35]. On the other hand, activation of MMPs is also involved in CCL3-mediated cell motility [36]. In this study, we found that while CCL3 induced MMP-2 expression and secretion in human chondrosarcoma cells, treatment of cells with the MMP-2 inhibitor reduced CCL3-induced cell migration. Moreover, inhibition of CCL3-enhanced MMP-2 protein expression, and siRNA knockdown of CCL-3 significantly suppressed CCL3-induced migration. Therefore, MMP-2 may be the CCL3-responsive mediator, and may degrade the ECM leading to subsequent cancer migration and metastasis.

AMPK is a heterotrimeric serine/threonine kinase composed of a catalytic subunit, α, and regulatory β and γ subunits [37]. Previous studies have shown that AMPK is involved in the CCL3 signaling pathway [38]. We observed that the AMPK inhibitors, namely, Ara A and compound C, antagonize CCL3-mediated cancer migration and MMP-2 expression, suggesting that AMPK activation is an obligatory event in CCL3-induced migration activity in these cells. We attempted to determine the identity of the catalytic subunit of AMPKα1 or AMPKα2, which mediates CCL3 signaling in human chondrosarcoma cells. We found that siRNA against both AMPKα1 and AMPKα2 reduce CCL3-mediated cancer migration, implying that AMPKα1 and AMPKα2 are involved in CCL3-induced migration activity. While adiponectin-mediated prostate cancer migration has been reported to be mediated by AMPKα1 but not AMPKα2 activation, [39] both AMPKα1 and AMPKα2 activation have been reported to mediate adiponectin-induced cell metastasis in human chondrosarcoma [30]. These data indicate that AMPKα1 and AMPKα2 are important for metastasis of human chondrosarcoma. Since it has also been reported that AMPK interacts with p38 to regulate cell motility in human chondrosarcoma [30], we examined the potential role of p38 in the signaling pathway of CCL3-induced migration activity. Pre-treatment of chondrosarcoma cells for 30 min with SB203580 or transfection with the p38 mutant for 24 h markedly attenuated the CCL3-induced migration activity and MMP-2 expression. In addition, we observed that treatment of chondrosarcoma cells with CCL3 induced increased p38 phosphorylation. These effects were inhibited by Ara A or compound C. In contrast, the p38 inhibitor did not affect CCL3-promoted AMPK phosphorylation, indicating the involvement of AMPK-dependent p38 activation in CCL3-mediated migration and MMP-2 expression.

Tumor metastasis is the spread of tumor cells from a primary tumor to colonize other sites of the body. Invasion, intravasation, extravasation through the circulatory system, colonization, and finally angiogenesis at a distant site are the most common features of tumor metastasis. Because of the prevalence of distant metastasis in patients with chondrosarcoma, prognosis is generally very poor and the development of anti-metastatic therapy could be useful for these patients. Here, we found that CCL3 induced MMP-2 expression and subsequently promoted migration in human chondrosarcoma through activation of the CCR5 receptor, AMPK, p38, and NF-κB signaling pathways (Figure 7C). These observations may provide a better understanding of the mechanisms of metastasis and may lead to the development of effective therapies for chondrosarcoma.

## Competing interest

All authors have no financial or personal relationships with other people or organizations that could inappropriately influence our work.

## Authors’ contributions

Performed the experiments: CJH, MHW, CYC. Analyzed the data: MHW, CYC HTH. Contributed reagents/materials/analysis tools: HHT. Wrote the paper: MHW; CHT. All authors read and approved the final manuscript.

## Supplementary Material

Additional file 1: Figure S1 AMPKα1 or AMPKα2 siRNA inhibited AMPKα1 or AMPKα2 expression. JJ012 cells were transfected with AMPKα1 or AMPKα2 siRNA for 24 h, the AMPKα1 or AMPKα2 expression was examined by western blotting. **Figure S2.** CCL3 and MMP-2 expression in chondrocyte and chondrosarcoma. Western blotting results of CCL3 and MMP-2 expression in chondrocytes and chondrosarcomas. **Figure S3.** CCL3 did not induce cell migration in primary chondrocytes. Primary chondrocytes were incubated with CCL3 for 24 h, and *in vitro* migration was measured by Transwell. The results are expressed as the mean ± SE. **Figure S4.** JJ012 expressed high level of CCL3 than chondrocytes. The protein levels of CCL3 in JJ012 cells and primary chondrocytes was measured by western blotting. **Figure S5.** CCR5, AMPK, and p38 signaling pathways are involved in CCL3-induced NF-κB activation. JJ012 cells were pretreated with CCR5 mAb, Met-RANTES, Ara A, compound C, SB203580, PDTC, and TPCK for 30 min (A) or were transfected with control siRNA, AMPKα1 siRNA, AMPKα2 siRNA, p38 mutant, IKKα mutant, or IKKβ mutant (B) before exposure to CCL3. NF-κB luciferase activity was measured, and the results were normalized to the β-galactosidase activity and expressed as the mean ± SE for three independent experiments performed in triplicate. *P < 0.05 compared with control. ^#^*P* < 0.05 compared with CCL3-treated group. Click here for file

## References

[B1] Terek RM Schwartz GK Devaney K Glantz L Mak S Healey JH Albino AP Chemotherapy and P-glycoprotein expression in chondrosarcoma J Orthop Res 1998 16 585 590 9820282 10.1002/jor.1100160510

[B2] Fidler IJ The organ microenvironment and cancer metastasis Differentiation 2002 70 498 505 12492492 10.1046/j.1432-0436.2002.700904.x

[B3] Wu MH Lo JF Kuo CH Lin JA Lin YM Chen LM Tsai FJ Tsai CH Huang CY Tang CH Endothelin-1 promotes MMP-13 production and migration in human chondrosarcoma cells through FAK/PI3K/Akt/mTOR pathways J Cell Physiol 2012 227 3016 3026 21959927 10.1002/jcp.23043

[B4] Egeblad M Werb Z New functions for the matrix metalloproteinases in cancer progression Nat Rev Cancer 2002 2 161 174 11990853 10.1038/nrc745

[B5] Kerkela E Saarialho-Kere U Matrix metalloproteinases in tumor progression: focus on basal and squamous cell skin cancer Exp Dermatol 2003 12 109 125 12702139 10.1034/j.1600-0625.2003.120201.x

[B6] Chu CY Cha ST Chang CC Hsiao CH Tan CT Lu YC Jee SH Kuo ML Involvement of matrix metalloproteinase-13 in stromal-cell-derived factor 1 alpha-directed invasion of human basal cell carcinoma cells Oncogene 2007 26 2491 2501 17099730 10.1038/sj.onc.1210040

[B7] Hou CH Hsiao YC Fong YC Tang CH Bone morphogenetic protein-2 enhances the motility of chondrosarcoma cells via activation of matrix metalloproteinase-13 Bone 2009 44 233 242 19038372 10.1016/j.bone.2008.09.021

[B8] Tsou HK Chen HT Hung YH Chang CH Li TM Fong YC Tang CH HGF and c-Met interaction promotes migration in human chondrosarcoma cells PLoS One 2013 8 e53974 23320110 10.1371/journal.pone.0053974 PMC3540013

[B9] Kulbe H Levinson NR Balkwill F Wilson JL The chemokine network in cancer–much more than directing cell movement Int J Dev Biol 2004 48 489 496 15349823 10.1387/ijdb.041814hk

[B10] Schall TJ Bacon K Camp RD Kaspari JW Goeddel DV Human macrophage inflammatory protein alpha (MIP-1 alpha) and MIP-1 beta chemokines attract distinct populations of lymphocytes J Exp Med 1993 177 1821 1826 7684437 10.1084/jem.177.6.1821 PMC2191042

[B11] Bennouna S Bliss SK Curiel TJ Denkers EY Cross-talk in the innate immune system: neutrophils instruct recruitment and activation of dendritic cells during microbial infection J Immunol 2003 171 6052 6058 14634118 10.4049/jimmunol.171.11.6052

[B12] Nakasone Y Fujimoto M Matsushita T Hamaguchi Y Huu DL Yanaba M Sato S Takehara K Hasegawa M Host-derived MCP-1 and MIP-1alpha regulate protective anti-tumor immunity to localized and metastatic B16 melanoma Am J Pathol 2012 180 365 374 22037251 10.1016/j.ajpath.2011.09.005

[B13] Arabzadeh A Chan C Nouvion AL Breton V Benlolo S Demarte L Turbide C Brodt P Ferri L Beauchemin N Host-related carcinoembryonic antigen cell adhesion molecule 1 promotes metastasis of colorectal cancer Oncogene 2013 32 849 860 22469976 10.1038/onc.2012.112

[B14] Wu Y Li YY Matsushima K Baba T Mukaida N CCL3-CCR5 axis regulates intratumoral accumulation of leukocytes and fibroblasts and promotes angiogenesis in murine lung metastasis process J Immunol 2008 181 6384 6393 18941229 10.4049/jimmunol.181.9.6384

[B15] Janciauskiene S Wright HT Inflammation, antichymotrypsin, and lipid metabolism: autogenic etiology of Alzheimer’s disease Bioessays 1998 20 1039 1046 10048303 10.1002/(SICI)1521-1878(199812)20:12<1039::AID-BIES10>3.0.CO;2-Z

[B16] Yin K Tang C Inflammation, lipid metabolism dysfunction, and hypertension: active research fields in atherosclerosis-related cardiovascular disease in China Sci China Life Sci 2011 54 976 979 22038011 10.1007/s11427-011-4225-3

[B17] Hursting SD Digiovanni J Dannenberg AJ Azrad M Leroith D Demark-Wahnefried W Kakarala M Brodie A Berger NA Obesity, energy balance, and cancer: new opportunities for prevention Cancer Prev Res (Phila) 2012 5 1260 1272 23034147 10.1158/1940-6207.CAPR-12-0140 PMC3641761

[B18] Bijland S Mancini SJ Salt IP Role of AMP-activated protein kinase in adipose tissue metabolism and inflammation Clin Sci (Lond) 2013 124 491 507 23298225 10.1042/CS20120536

[B19] O’Neill HM AMPK and exercise: glucose uptake and insulin sensitivity Diabetes Metab J 2013 37 1 21 23441028 10.4093/dmj.2013.37.1.1 PMC3579147

[B20] Tang CH Lu ME Adiponectin increases motility of human prostate cancer cells via adipoR, p38, AMPK, and NF-kappaB pathways Prostate 2009 69 1781 1789 19676095 10.1002/pros.21029

[B21] Tan TW Yang WH Lin YT Hsu SF Li TM Kao ST Chen WC Fong YC Tang CH Cyr61 increases migration and MMP-13 expression via alphavbeta3 integrin, FAK, ERK and AP-1-dependent pathway in human chondrosarcoma cells Carcinogenesis 2009 30 258 268 19126648 10.1093/carcin/bgn284

[B22] Wu MH Chen LM Hsu HH Lin JA Lin YM Tsai FJ Tsai CH Huang CY Tang CH Endothelin-1 enhances cell migration through COX-2 up-regulation in human chondrosarcoma Biochim Biophys Acta 2013 1830 3355 3364 23523690 10.1016/j.bbagen.2013.03.014

[B23] Tang CH Hsu CJ Fong YC The CCL5/CCR5 axis promotes interleukin-6 production in human synovial fibroblasts Arthritis Rheum 2010 62 3615 3624 20862675 10.1002/art.27755

[B24] Huang CY Chen SY Tsai HC Hsu HC Tang CH Thrombin induces epidermal growth factor receptor transactivation and CCL2 expression in human osteoblasts Arthritis Rheum 2012 64 3344 3354 22674286 10.1002/art.34557

[B25] Hou CH Chiang YC Fong YC Tang CH WISP-1 increases MMP-2 expression and cell motility in human chondrosarcoma cells Biochem pharmacol 2011 81 1286 1295 21453685 10.1016/j.bcp.2011.03.016

[B26] Roberti MP Arriaga JM Bianchini M Quinta HR Bravo AI Levy EM Mordoh J Barrio MM Protein expression changes during human triple negative breast cancer cell line progression to lymph node metastasis in a xenografted model in nude mice Cancer Biol Ther 2012 13 1123 1140 22825326 10.4161/cbt.21187 PMC3461818

[B27] Tan TW Lai CH Huang CY Yang WH Chen HT Hsu HC Fong YC Tang CH CTGF enhances migration and MMP-13 up-regulation via alphavbeta3 integrin, FAK, ERK, and NF-kappaB-dependent pathway in human chondrosarcoma cells J Cell Biochem 2009 107 345 356 19301259 10.1002/jcb.22132

[B28] Zucchetto A Benedetti D Tripodo C Bomben R Dal Bo M Marconi D Bossi F Lorenzon D Degan M Rossi FM CD38/CD31, the CCL3 and CCL4 chemokines, and CD49d/vascular cell adhesion molecule-1 are interchained by sequential events sustaining chronic lymphocytic leukemia cell survival Cancer research 2009 69 4001 4009 19383907 10.1158/0008-5472.CAN-08-4173

[B29] Kim Y Regulation of cell proliferation and migration in glioblastoma: new therapeutic approach Front Oncol 2013 3 53 23508546 10.3389/fonc.2013.00053 PMC3600576

[B30] Chiu YC Shieh DC Tong KM Chen CP Huang KC Chen PC Fong YC Hsu HC Tang CH Involvement of AdipoR receptor in adiponectin-induced motility and alpha2beta1 integrin upregulation in human chondrosarcoma cells Carcinogenesis 2009 30 1651 1659 19549705 10.1093/carcin/bgp156

[B31] Omoike OI Teague RM Benedict SH Chan MA MIP-1alpha induces binding of nuclear factors to the kappaB DNA element in human B cells MCBRC 2000 4 15 19 11152622 10.1006/mcbr.2000.0247

[B32] Fong YC Yang WH Hsu SF Hsu HC Tseng KF Hsu CJ Lee CY Scully SP 2-methoxyestradiol induces apoptosis and cell cycle arrest in human chondrosarcoma cells J Orthop Res 2007 25 1106 1114 17415781 10.1002/jor.20364

[B33] Menu E De Leenheer E De Raeve H Coulton L Imanishi T Miyashita K Van Valckenborgh E Van Riet I Van Camp B Horuk R Role of CCR1 and CCR5 in homing and growth of multiple myeloma and in the development of osteolytic lesions: a study in the 5TMM model Clin Exp Metastasis 2006 23 291 300 17086356 10.1007/s10585-006-9038-6

[B34] Deryugina EI Quigley JP Matrix metalloproteinases and tumor metastasis Cancer Metastasis Rev 2006 25 9 34 16680569 10.1007/s10555-006-7886-9

[B35] Scherer RL McIntyre JO Matrisian LM Imaging matrix metalloproteinases in cancer Cancer Metastasis Rev 2008 27 679 690 18465089 10.1007/s10555-008-9152-9

[B36] Repeke CE Ferreira SB Jr Claudino M Silveira EM de Assis GF Avila-Campos MJ Silva JS Garlet GP Evidences of the cooperative role of the chemokines CCL3, CCL4 and CCL5 and its receptors CCR1+ and CCR5+ in RANKL + cell migration throughout experimental periodontitis in mice Bone 2010 46 1122 1130 20053385 10.1016/j.bone.2009.12.030

[B37] Gruzman A Babai G Sasson S Adenosine monophosphate-activated protein kinase (AMPK) as a new target for antidiabetic drugs: a review on metabolic, pharmacological and chemical considerations Rev Diabet Stud 2009 6 13 36 19557293 10.1900/RDS.2009.6.13 PMC2712919

[B38] Taddei SR Queiroz CM Jr Moura AP Andrade I Jr Garlet GP Proudfoot AE Teixeira MM da Silva TA The effect of CCL3 and CCR1 in bone remodeling induced by mechanical loading during orthodontic tooth movement in mice Bone 2013 52 259 267 23059626 10.1016/j.bone.2012.09.036

[B39] Chen YJ Wei YY Chen HT Fong YC Hsu CJ Tsai CH Hsu HC Liu SH Tang CH Osteopontin increases migration and MMP-9 up-regulation via alphavbeta3 integrin, FAK, ERK, and NF-kappaB-dependent pathway in human chondrosarcoma cells J Cell Physiol 2009 221 98 108 19475568 10.1002/jcp.21835

