# Supplementary material for: AMP-activated protein kinase activation mediates CCL3-induced cell migration and matrix metalloproteinase-2 expression in human chondrosarcoma
Source: Cell Commun Signal. 2013 Sep 18;11:68. doi: 10.1186/1478-811X-11-68 (PMC3851317; doi:10.1186/1478-811X-11-68)
Supplement: Additional file 1: Figure S1 — AMPKα1 or AMPKα2 siRNA inhibited AMPKα1 or AMPKα2 expression. JJ012 cells were transfected with AMPKα1 or AMPKα2 siRNA for 24 h, the AMPKα1 or AMPKα2 expression was examined by western blotting. Figure S2. CCL3 and MMP-2 expression in chondrocyte and chondrosarcoma. Western blotting results of CCL3 and MMP-2 expression in chondrocytes and chondrosarcomas. Figure S3. CCL3 did not induce cell migration in primary chondrocytes. Primary chondrocytes were incubated with CCL3 for 24 h, and in vitro migration was measured by Transwell. The results are expressed as the mean ± SE. Figure S4. JJ012 expressed high level of CCL3 than chondrocytes. The protein levels of CCL3 in JJ012 cells and primary chondrocytes was measured by western blotting. Figure S5. CCR5, AMPK, and p38 signaling pathways are involved in CCL3-induced NF-κB activation. JJ012 cells were pretreated with CCR5 mAb, Met-RANTES, Ara A, compound C, SB203580, PDTC, and TPCK for 30 min (A) or were transfected with control siRNA, AMPKα1 siRNA, AMPKα2 siRNA, p38 mutant, IKKα mutant, or IKKβ mutant (B) before exposure to CCL3. NF-κB luciferase activity was measured, and the results were normalized to the β-galactosidase activity and expressed as the mean ± SE for three independent experiments performed in triplicate. *P < 0.05 compared with control. #P < 0.05 compared with CCL3-treated group. [file 1478-811X-11-68-S1.doc]

**Supplementary** **Data**

**
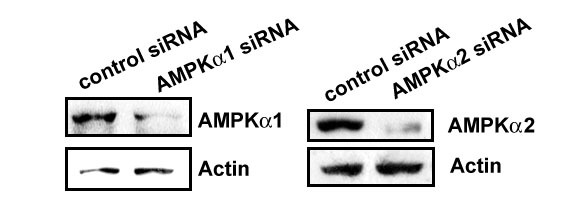
**

**Fig. S1. AMPKα1 or AMPKα2 siRNA inhibited AMPKα1 or AMPKα2 expression.** JJ012 cells were transfected with AMPKα1 or AMPKα2 siRNA for 24 h, the AMPKα1 or AMPKα2 expression was examined by western blotting.

**
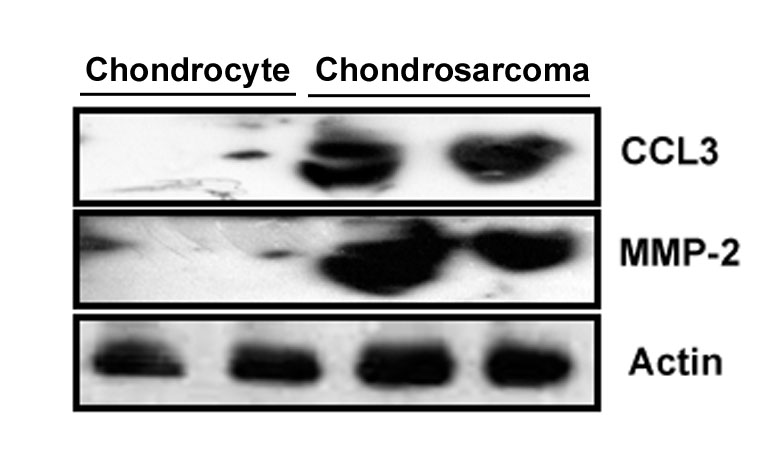
**

**Fig. S2. CCL3 and MMP-2 expression in chondrocyte and chondrosarcoma.** Western blotting results of CCL3 and MMP-2 expression in chondrocytes and chondrosarcomas.

**Fig. S3. CCL3 did not induce cell migration in primary chondrocytes.** Primary chondrocytes were incubated with CCL3 for 24 h, and *in vitro* migration was measured by Transwell. The results are expressed as the mean ± SE.

**
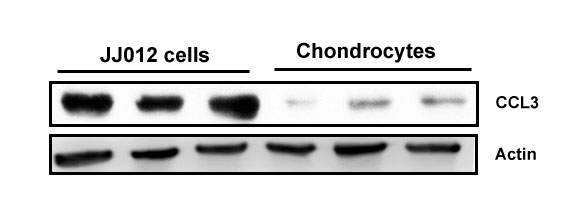
**

**Fig. S4. JJ012 expressed high level of CCL3 than chondrocytes.** The protein levels of CCL3 in JJ012 cells and primary chondrocytes was measured by western blotting.

**
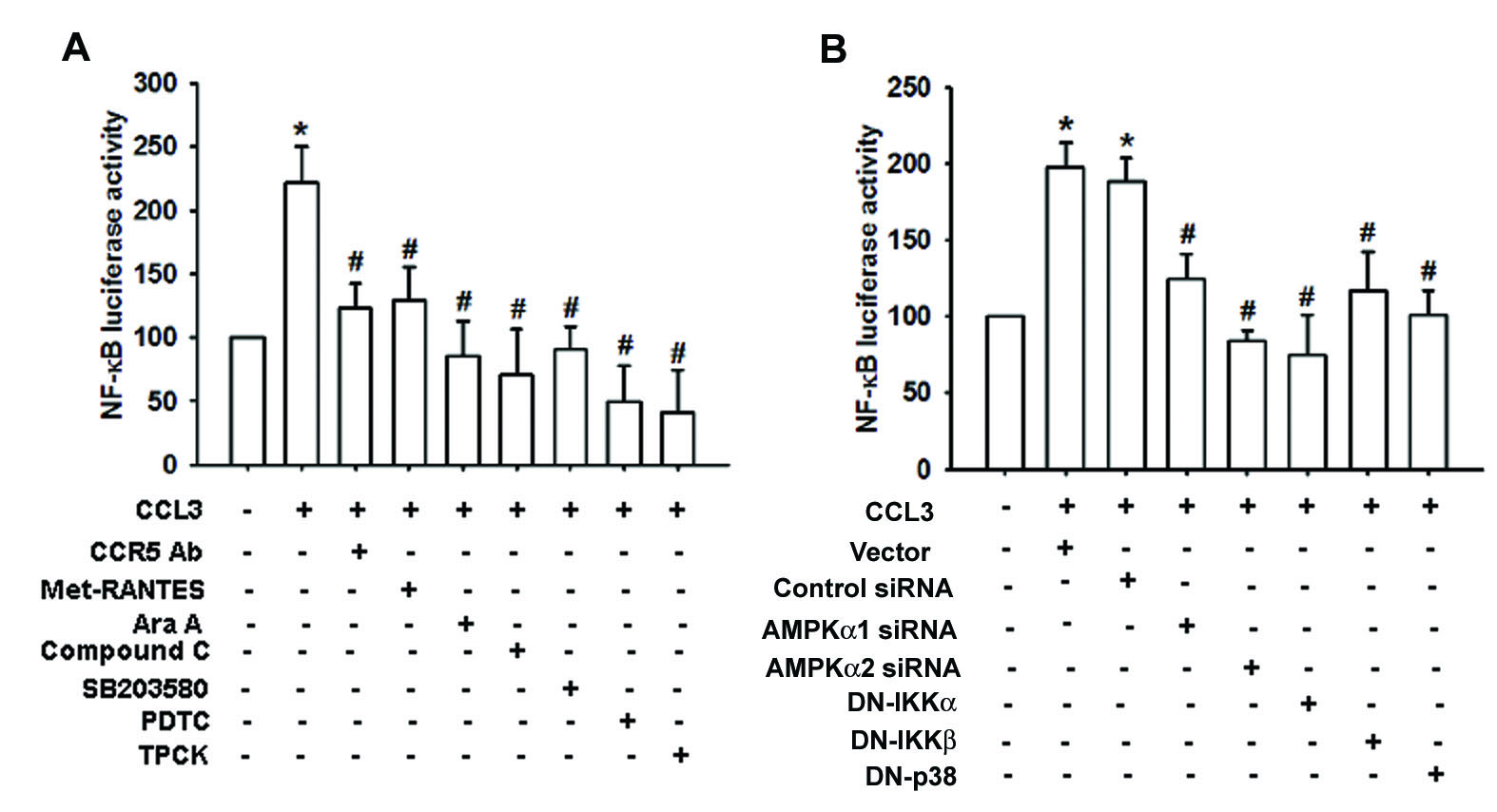
**

**Fig. S5. CCR5, AMPK, and p38 signaling pathways are involved in CCL3-induced NF-κB activation.** JJ012 cells were pretreated with CCR5 mAb, Met-RANTES, Ara A, compound C, SB203580, PDTC, and TPCK for 30 min **(A)** or were transfected with control siRNA, AMPKα1 siRNA, AMPKα2 siRNA, p38 mutant, IKKα mutant, or IKKβ mutant **(B)** before exposure to CCL3. NF-κB luciferase activity was measured, and the results were normalized to the β-galactosidase activity and expressed as the mean ± SE for three independent experiments performed in triplicate. **P* < 0.05 compared with control. ^#^*P* < 0.05 compared with CCL3-treated group.
